# Classification of idiopathic interstitial pneumonias using anti–myxovirus resistance-protein 1 autoantibody

**DOI:** 10.1038/srep43201

**Published:** 2017-02-23

**Authors:** Yoshimasa Hamano, Hiroshi Kida, Shoichi Ihara, Akihiro Murakami, Masahiro Yanagawa, Ken Ueda, Osamu Honda, Lokesh P. Tripathi, Toru Arai, Masaki Hirose, Toshimitsu Hamasaki, Yukihiro Yano, Tetsuya Kimura, Yasuhiro Kato, Hyota Takamatsu, Tomoyuki Otsuka, Toshiyuki Minami, Haruhiko Hirata, Koji Inoue, Izumi Nagatomo, Yoshito Takeda, Masahide Mori, Hiroyoshi Nishikawa, Kenji Mizuguchi, Takashi Kijima, Masanori Kitaichi, Noriyuki Tomiyama, Yoshikazu Inoue, Atsushi Kumanogoh

**Affiliations:** 1 Department of Respiratory Medicine, Allergy and Rheumatic Diseases, Osaka University Graduate School of Medicine, 2-2 Yamadaoka, Suita City, Osaka 565-0871, Japan; 2 AMED, CREST, Suita City, Osaka 565-0871, Japan; 3 Medical & Biological Laboratories Co., Ltd., Ina Laboratory, 1063-103 Terasawaoka, Ina City, Nagano 396-0002, Japan; 4 Department of Radiology, Osaka University Graduate School of Medicine, 2-2 Yamadaoka, Suita City, Osaka 565-0871, Japan; 5 National Institutes of Biomedical Innovation, Health and Nutrition, 7-6-8 Saitoasagi, Ibaraki City, Osaka 567-0085, Japan; 6 National Hospital Organization Kinki-Chuo Chest Medical Center, 1180 Nagasone-Cho, Kita-Ku, Sakai City, Osaka 591-8555, Japan; 7 Office of Biostatistics and Data Management, National Cerebral and Cardiovascular Center, 5-7-1 Fujishirodai, Suita City, Osaka 565-8565, Japan; 8 National Hospital Organization Toneyama National Hospital, 5-1-1 Toneyama, Toyonaka City, Osaka 560-8552, Japan; 9 Department of Immunopathology, WPI Immunology Frontier Research Center, Osaka University, Yamadaoka 3-1, Suita City, Osaka 565-0871, Japan; 10 Department of Experimental Immunology, WPI Immunology Frontier Research Center, Osaka University, Yamadaoka 3-1, Suita City, Osaka 565-0871, Japan

## Abstract

Chronic fibrosing idiopathic interstitial pneumonia (IIP) can be divided into two main types: idiopathic pulmonary fibrosis (IPF), a steroid-resistant and progressive disease with a median survival of 2–3 years, and idiopathic non-specific interstitial pneumonia (INSIP), a steroid-sensitive and non-progressive autoimmune disease. Although the clinical courses of these two diseases differ, they may be difficult to distinguish at diagnosis. We performed a comprehensive analysis of serum autoantibodies from patients definitively diagnosed with IPF, INSIP, autoimmune pulmonary alveolar proteinosis, and sarcoidosis. We identified disease-specific autoantibodies and enriched KEGG pathways unique to each disease, and demonstrated that IPF and INSIP are serologically distinct. Furthermore, we discovered a new INSIP-specific autoantibody, anti–myxovirus resistance-1 (MX1) autoantibody. Patients positive for anti-MX1 autoantibody constituted 17.5% of all cases of chronic fibrosing IIPs. Notably, patients rarely simultaneously carried the anti-MX1 autoantibody and the anti–aminoacyl-transfer RNA synthetase autoantibody, which is common in chronic fibrosing IIPs. Because *MX1* is one of the most important interferon-inducible anti-viral genes, we have not only identified a new diagnostic autoantibody of INSIP but also obtained new insight into the pathology of INSIP, which may be associated with viral infection and autoimmunity.

According to the international consensus classification of the American Thoracic Society (ATS) and European Respiratory Society (ERS), based on multi-disciplinary diagnosis (MDD), chronic fibrosing idiopathic interstitial pneumonia (IIP) includes two diseases, idiopathic pulmonary fibrosis (IPF) and idiopathic nonspecific interstitial pneumonia (INSIP)^1^,^2^. IPF is a steroid-resistant fatal lung disease that is characterized by worsening dyspnea and progressive loss of lung function. By contrast, INSIP may be a steroid-sensitive disease associated with a more favorable prognosis^3^,^4^,^5^. Although the clinical courses of these diseases differ, discrimination between IPF and INSIP at diagnosis may be difficult^6^,^7^. Moreover, the distinction between IPF and INSIP at the molecular level remains ambiguous^8^,^9^. Recent evidence revealed that even patients diagnosed with IPF follow different clinical courses^5^,^10^. Moreover, other conditions, such as chronic hypersensitivity pneumonitis or interstitial pneumonia associated with collagen vascular disease (CVD), are often confused with IPF or INSIP^2^,^11^. Many patients diagnosed with IIPs have clinical features that suggest underlying autoimmune processes but do not meet established criteria for CVDs, such as American College of Rheumatology criteria. Today, ERS/ATS proposed the term, interstitial pneumonia with autoimmune features (IPAF), and provides the classification criteria^12^,^13^. Therefore, identification of new autoantibody that can clearly distinguish a unique subgroup of patients within chronic fibrosing IIPs would facilitate accurate classification based on autoimmunity and expand the concept of IPAF.

The presence of autoantibodies may identify patients with specific autoimmune syndromes associated with interstitial lung disease. For example, polymyositis/dermatomyositis is a chronic inflammatory disorder with heterogeneous clinical features, including varying degrees of skin manifestations, myositis, and interstitial pneumonia. An autoantibody against melanoma differentiation–associated gene-5 (MDA5) can be used to distinguish a unique subgroup of patients with polymyositis/dermatomyositis who exhibit clinically amyopathic dermatomyositis, particularly when complicated by acute progressive interstitial lung disease^14^. Recently, anti–aminoacyl tRNA synthetase (ARS) autoantibody was reported to effectively distinguish a subgroup of patients with idiopathic inflammatory myopathy, called anti-synthetase syndrome, who typically have interstitial pneumonia, myositis, non-erosive arthritis, Raynaud’s phenomenon, fever, and mechanic’s hands. Anti-ARS autoantibodies are also present in 7–10% of patients with chronic IIPs who exhibited INSIP-like clinical characteristics^15^,^16^. Immune processes play a major role in the disease pathogenesis and progression of INSIP^5^,^17^; however, there is still no reliable method for using serum samples to identify and characterize immune processes unique to INSIP.

Protein arrays that enable detection of specific serum antibodies against over 8,000 targets randomly selected from throughout the human genome have been used to analyze immune responses in various diseases^18^,^19^,^20^. We hypothesized that identification of new autoantibodies or a repertoire of autoantibodies specifically associated with INSIP might serve as biomarkers capable of distinguishing a unique subgroup of patients with chronic fibrosing IIPs who share some clinical characteristics with patients with INSIP.

The type I interferon (IFN) system induces the expression of various antiviral proteins and IFN-inducible genes when activated in response to viral infection, including myxovirus resistance protein (MX) and MDA5. MX is a dynamin-like GTPase. Humans express two MX family proteins, MX1 and MX2, encoded by the *MX1* and *MX2* genes, respectively, on chromosome 21^21^. MX1 expression is elevated in infectious diseases and type I IFN–driven autoimmune diseases such as systemic lupus erythematosus^22^. However, no studies have reported an association between anti-MX1 autoantibodies and disease.

We conducted three independent studies to clarify and discover autoantibodies that could serve as biomarkers for differential diagnosis in patients with IIPs. First, in our discovery cohort, we tried to identify a group of autoantibodies specific to each of the four independent inflammatory lung diseases [IPF, INSIP, autoimmune pulmonary alveolar proteinosis (aPAP), and sarcoidosis] using a protein array to search for serum autoantibody signatures unique to IPF and INSIP. Second, we conducted a cross-sectional study to characterize patients with chronic fibrosing IIPs who were also positive for anti-MX1 autoantibody. Finally, we conducted a nested case–control study to elucidate the prognosis of anti-MX1 autoantibody–positive patients with chronic fibrosing IIPs.

## Results

### Identification of disease-specific immunogenic signatures of IPF, INSIP, sarcoidosis, and aPAP

**Figure 1:**
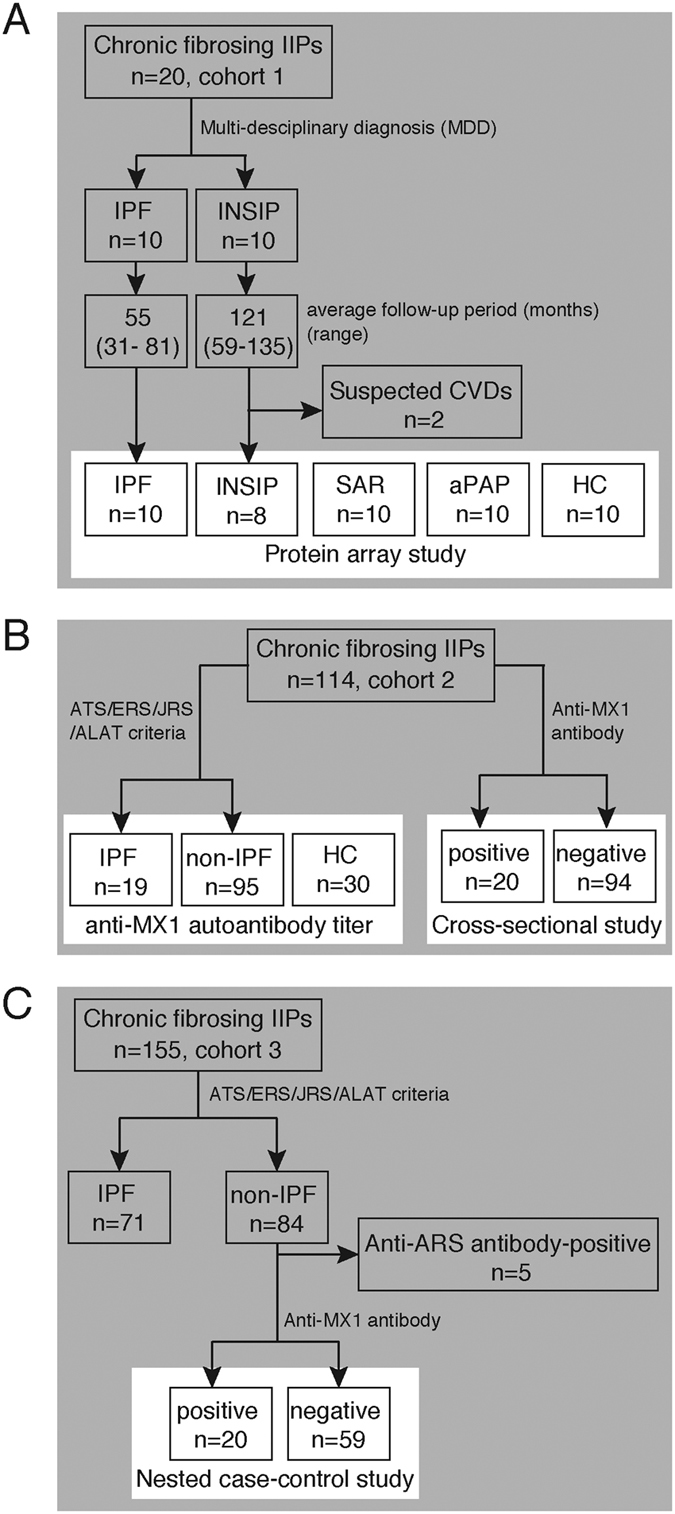
Three independent cohorts of chronic fibrosing IIPs were involved in this study. (**A**) Cohort 1 consisted of 10 IPF and 10 INSIP patients, who satisfied multi-disciplinary diagnosis (MDD). Surgical lung biopsy was performed on all patients. The average follow-up periods after diagnosis of IPF and INSIP were 55 (range, 31–81) and 121 (range, 59–135) months, respectively. During the follow-up period, two INSIP patients were highly suspected to have collagen vascular diseases (CVDs) and excluded from the study. The remaining 18 patients from cohort 1, 10 patients with sarcoidosis (SAR), 10 patients with autoimmune pulmonary alveolar proteinosis (aPAP), and 10 healthy controls (HCs) were included in the protein array study (white box). (**B**) Cohort 2 consisted of 114 patients with chronic fibrosing IIPs (IPF, n = 19; non-IPF, n = 95), who consecutively visited the Osaka University Hospital between February 2014 and October 2014. Serum anti-MX1 autoantibody was measured in all patients in cohort 2 and 30 healthy controls, and the cut-off value for anti-MX1 autoantibody was determined (white box, left). Using this cut-off value, cohort 2 was divided into anti-MX1 autoantibody–positive (n = 20) and –negative (n = 94) groups, and a cross-sectional study was performed (white box, right). (C) Cohort 3 consisted of 155 patients with chronic fibrosing IIPs registered at National Hospital Organization Kinki-Chuo Chest Medical Center between 2005 and 2009. Among 84 non-IPF patients in cohort 3, five patients positive for serum anti-ARS antibody were removed. The remaining 79 patients were divided into anti-MX1 autoantibody–positive and –negative groups, and a nested case–control study was performed (white box).

First, we sought to serologically characterize the four independent inflammatory lung diseases (IPF, INSIP, aPAP, and sarcoidosis) by comprehensively measuring serum autoantibodies using protein arrays (Table 1). Ten patients with IPF and INSIP (cohort 1), all of who received surgical lung biopsy and were definitively diagnosed by MDD according to ATS/ERS criteria, were included in the study (Fig. 1A). To confirm these diagnoses, we observed the patients for long periods (average: IPF, 55 months; INSIP, 121 months) to rule out the development of CVDs after the initial diagnosis. During the follow-up period, two patients who were initially diagnosed with INSIP but later proved to be with highly suspected CVDs were removed. None of the remaining eight patients with INSIP were classified as IPAF according to the ERS/ATS criteira^13^. Sarcoidosis is a distinct inflammatory lung disease for which the target antigen responsible for inflammation remains unknown. On the other hand, aPAP is caused by anti–GM-CSF autoantibody, and was included as a positive control to confirm the validity of our screening method (Fig. 1A). We calculated the antigenic score (*AS*) for each antigen from its protein array measurement as previously reported^18^,^19^. This method is designed to identify rare but clear events, corresponding to high-titer antigens that are only occasionally detected in tested sera. The *AS* of GM-CSF, the aPAP-specific autoantigen, was the highest of all antigens tested in this study, supporting the validity of *AS*. As we incrementally increased the *AS* threshold, we found that a threshold of *AS* ≥ 57.1 could resolve cohort-specific antigens, i.e., those that exhibited substantially higher responses in a particular disease cohort relative to the others. We identified 40, 51, 57, and 44 proteins specifically associated with IPF, INSIP, aPAP, and sarcoidosis, respectively (Table 2 and Supplementary Table S1). These included glycyl-tRNA synthetase (GARS), which exhibited the highest immunogenicity in INSIP relative to the other cohorts (*AS* = 151.6; Table 2); this was in agreement with a previous report that anti-GARS antibodies are detectable in the sera of patients with INSIP^23^. Furthermore, two related proteins, glutaminyl-tRNA synthetase (QARS) and methionyl-tRNA synthetase (MARS), also exhibited significantly enhanced immunogenicity in the INSIP cohort relative to other disease cohorts, consistent with the previously observed association between different anti-ARS antibodies and patients with INSIP^15^,^16^. These observations suggested that the significantly antigenic proteins identified by our integrative approach closely reflected the immunogenic profiles of inflammatory lung diseases *in vivo*.

Next, we investigated proteins associated with specific cohorts for the enrichment of specific KEGG-pathway associations. Analysis of IPF-, INSIP-, aPAP-, and sarcoidosis-associated proteins revealed enrichment (*p* ≤ 0.05) of 7, 13, 7, and 2 KEGG pathways, respectively (Table 3). Notably, there was no overlap in the enriched KEGG pathways associated with the four cohorts, except for “Influenza A” (hsa05164), which was associated with both INSIP and aPAP (*p* = 0.033 and *p* = 0.045, respectively). These data suggested that the *AS* ≥ 57.1 threshold successfully distinguished specific functional themes associated with the physiological and immune responses in individual disease cohorts. In addition, these results provided the first evidence that IPF and INSIP, diagnosed by ATS/ERS criteria, are serologically distinct entities.

### Identification of an anti-MX1 autoantibody as a candidate to distinguish a subgroup of patients with chronic fibrosing IIPs

**Figure 2:**
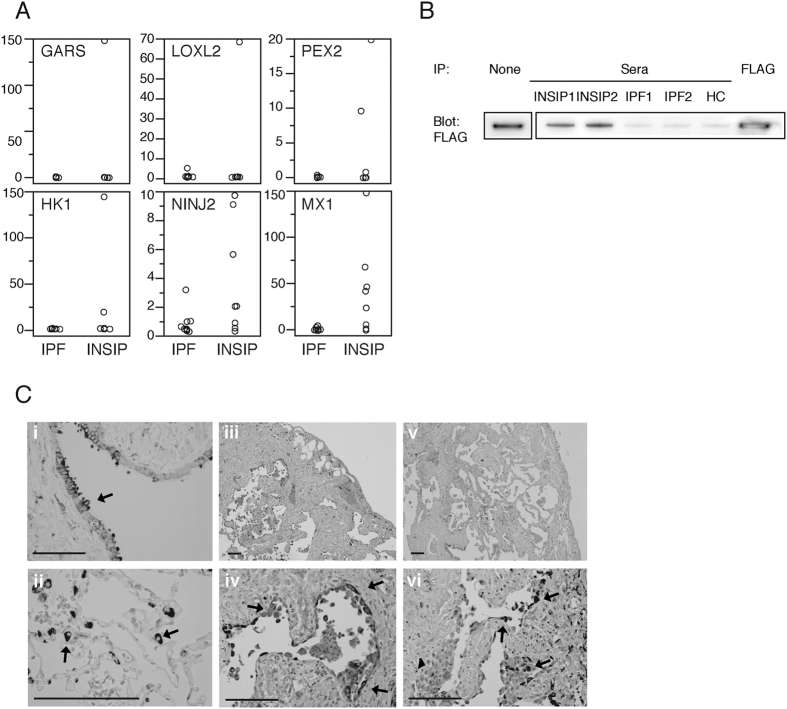
(**A**) Top six INSIP-specific autoantibodies identified in protein arrays. Distributions of normalized values across all arrays are shown. IPF, n = 10; INSIP, n = 8. Abbreviations: GARS, glycyl-tRNA synthetase; LOXL2, lysyl oxidase–like 2; PEX2, peroxisomal biogenesis factor 2; HK1, hexokinase 1; NINJ2, ninjurin 2; MX1, myxovirus resistance protein 1. (**B**) Lysates from HEK293 cells overexpressing FLAG-tagged MX1 were immunoprecipitated using sera from patients with INSIP, patients with IPF, or healthy control (HC) subjects. (**C**) Immunohistochemical analysis of MX1 in the lungs of control individuals (i,ii), patients with IPF (iii,iv), and patients with INSIP (v,vi). Scale bars denote 200 μm.

Next, we sought to identify an autoantibody that distinguishes a subgroup of patients within chronic fibrosing IIPs. As shown in Fig. 2A, even the autoantibodies with the highest *AS* scores did not react with sera from all patients with INSIP (Fig. 2A). Therefore, we focused on the MX1 autoantigen, which was present at highly elevated levels in many patients with INSIP (Fig. 2A). The presence of anti-MX1 autoantibody in patients with INSIP was confirmed by immunoprecipitation assay. Sera from patients with INSIP immunoprecipitated recombinant FLAG-tagged MX1 expressed in HEK293 cells, whereas sera from healthy controls (HCs) and patients with IPF did not (Fig. 2B). Immunohistochemistry revealed that in normal lung, MX1 was expressed in Clara cells, alveolar type II pneumocytes, and alveolar macrophages (Fig. 2Ci,ii). By contrast, in the lungs of IPF (Fig. 2Ciii,iv) and INSIP patients (Fig. 2Cv,vi), MX1 was upregulated and localized in hyperplastic type II pneumocytes and aggregated macrophages. Either the distribution or the intensity of MX1 expression was not different between IPF and INSIP.

### Establishment of an enzyme-linked immunosorbent assay (ELISA) for the measurement of serum anti-MX1 antibody production

**Figure 3:**
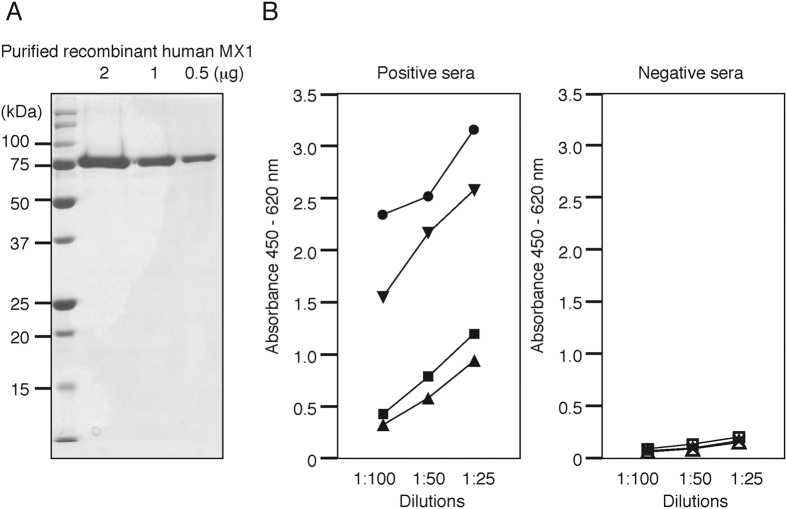
(**A**) SDS-PAGE of purified recombinant human MX1. (**B**) Patients’ sera were diluted 1:100, 1:50, or 1:25. In the ELISA system we developed, reactivities increased in a concentration-dependent manner in four anti-MX1 autoantibody–positive sera (left panel), but not in three anti-MX1 autoantibody–negative sera (right panel).

To develop an ELISA system for the detection of anti-MX1 autoantibodies, recombinant His-tagged human MX1 protein was expressed using a baculovirus system and purified by nickel ion–affinity chromatography (Fig. 3A). To determine whether this system could detect serum anti-MX1 autoantibodies in a concentration-dependent manner, we assayed 1:100, 1:50, and 1:25 dilutions of serum from four anti-MX1 autoantibody–positive patients and three autoantibody-negative patients. All of the anti-MX1 autoantibody–positive sera exhibited increased reactivity in a concentration-dependent manner. However, the reactivities of the anti-MX1 autoantibody–negative sera were only marginally altered (Fig. 3B). Our ELISA system was highly reproducible, as demonstrated by the low intra- and inter-assay coefficients of variation (CVs), which were both less than 10%.

### Prevalence and clinical characteristics of anti-MX1 autoantibody–positive patients with IIPs

**Figure 4:**
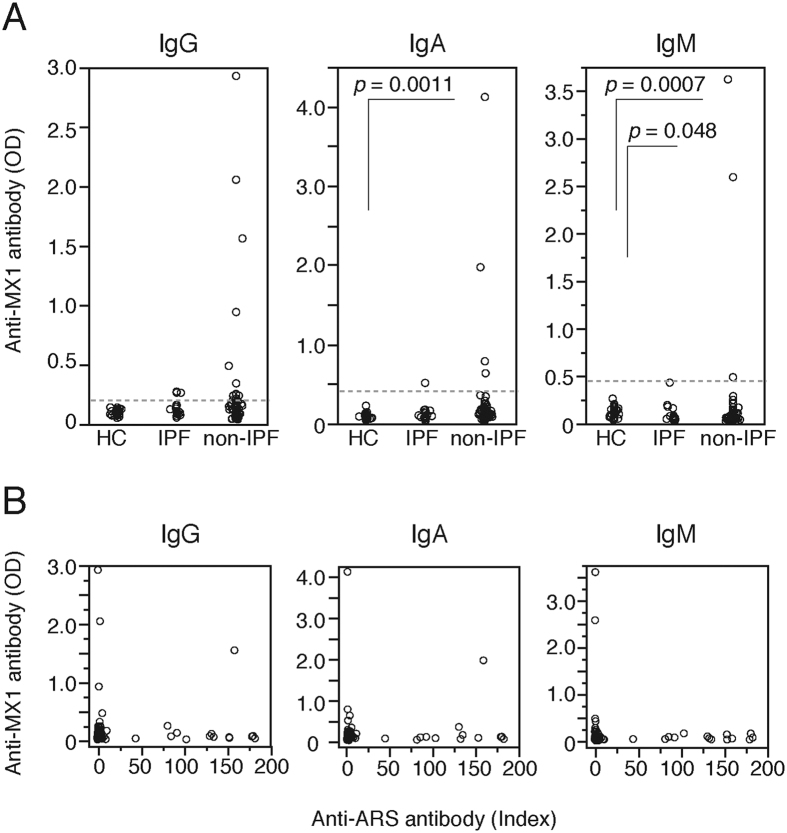
(**A**) Titer distributions of IgG, IgA, and IgM anti-MX1 autoantibodies, measured by ELISA. Healthy controls (HCs), n = 30; patients with IPF, n = 19; patients without IPF, n = 95. Pairwise comparisons among groups were performed by the Steel–Dwass procedure. The dotted line shows the cut-off value, which was determined as the mean absorbance +6 SDs of values in HCs. (**B**) Titers of anti-ARS autoantibodies (Jo-1, PL-7, PL-12, EJ, and KS) were plotted against those of IgG, IgA, and IgM anti-MX1 autoantibodies.

We consecutively enrolled 114 patients with chronic fibrosing IIPs who visited the outpatient office of Osaka University Hospital between February 2014 and October 2014 (cohort 2) and measured serum anti-MX1 (IgG, IgA, and IgM type) and anti-ARS autoantibody concentrations by ELISA. Anti-ARS is a well-characterized autoantibody present in about 10% of patients with chronic fibrosing IIPs^15^,^16^. In this cohort, 19 patients, including four patients who received surgical lung biopsy, were classified as having IPF according to the 2011 ATS/ERS/JRS/ALAT criteria, whereas the remaining 95 patients, including seven diagnosed with INSIP following surgical lung biopsy, were classified as non-IPF. For all types of anti-MX1 autoantibodies, very high levels of autoantibody production were detected only in the non-IPF group (Fig. 4A). Cut-off values for anti-MX1 autoantibody were defined as the mean values plus six standard deviations (SDs) from 30 healthy controls. Using this definition, 20 patients with chronic fibrosing IIPs (17.5%, 95% CI: 11.7–25.6%) were positive for either IgG, IgA, or IgM-type anti-MX1 autoantibody and classified as anti-MX1 autoantibody–positive patients (Fig. 4A). As shown in Fig. 4B, there was no relationship between the serum levels of anti-MX1 and anti-ARS autoantibodies. In addition, it was very rare for patients to have elevated levels of anti-MX1 and anti-ARS antibodies at the same time. In cohort 2, retrospectively, 33 patients could be classified as IPAF according to the ERS/ATS criteria^13^. Anti-MX1 autoantibody was detected in 7 (21.2%) of 33 IPAF patients, and 13 (16.0%) of 81 non-IPAF patients. The positivity of anti-MX1 autoantibody was not significantly associated with IPAF category (p = 0.59).

The cross-sectional study, in which we compared clinical characteristics between anti-MX1 autoantibody–positive (n = 20) and –negative (n = 94) patients with chronic fibrosing IIPs by univariable analysis, revealed that anti-MX1 autoantibody–positive status was associated with female sex and the absence of two high-resolution computed tomography (HRCT) findings, predominantly lower and predominantly peripheral distribution (Table 4). To clarify the results by removing the effects of unknown confounding factors, we performed multivariable logistic regression analysis. Because these two HRCT findings were simultaneously positive in 80/114 (70.2%) patients, we initially created a combined HRCT finding, predominantly lower and peripheral distribution, and included it in stepwise regression analysis with four other factors (gender, %D_LCO_, glucocorticoids, and % spared area in HRCT). We ultimately selected three factors (gender, %D_LCO_, and predominantly lower and peripheral distribution) as the variables for multivariable logistic regression analysis. This analysis revealed that anti-MX1 autoantibody–positive status among chronic fibrosing IIPs was associated with female sex and the absence of predominantly lower and peripheral distribution (Table 5).

### Prognosis of anti-MX1 autoantibody–positive patients with IIPs

**Figure 5:**
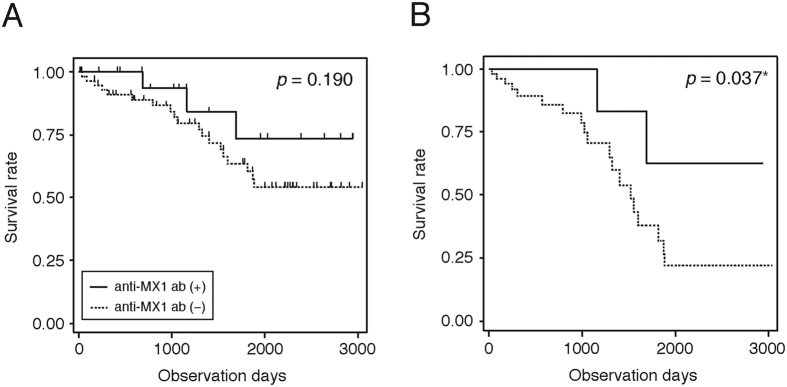
Kaplan–Meier curves of overall survivals of anti-MX1 antibody–positive (n = 20) and –negative (n = 59) non-IPF patients before (**A**) and after (**B**) the adjustment by staging based on the modified ILD-GAP index. Statistical comparison between the groups was performed by generalized (**A**) and stratified (**B**) Wilcoxon test, respectively.

To examine the prognosis of anti-MX1 autoantibody–positive patients with IIPs, we performed a nested case–control study. For this purpose, we used a retrospective cohort of patients with IIPs on whom bronchoalveolar lavage, pulmonary function test, and serum sampling were performed at the time of registration between 2005 and 2009 at National Hospital Organization Kinki-Chuo Chest Medical Center (cohort 3) (Fig. 1C). In this cohort, 71 patients, including 35 patients who underwent surgical lung biopsy, were classified as having IPF according to the 2011 ATS/ERS/JRS/ALAT criteria, and the remaining 84 patients, including 10 who were diagnosed with INSIP by surgical lung biopsy, were classified as non-IPF. In the non-IPF group, 20 patients were positive for anti-MX1 autoantibody, five (6.0%) were positive for anti-ARS autoantibody, and no patients were positive for both. We excluded five anti-ARS autoantibody–positive patients from the subsequent analysis and compared survival between anti-MX1 autoantibody–positive non-IPF (n = 20) and –negative non-IPF (n = 59) (Fig. 1C). At the time of registration, no clinical parameters listed in Supplementary Table S2 differed significantly between the two groups. Among non-IPF patients, those who were anti-MX1 autoantibody–positive had better prognosis than autoantibody-negative patients, but the difference was not statistically significant. However, after adjusting for staging using the modified interstitial lung disease gender–age–physiology (ILD-GAP) index, established as a prognostic predictor^24^,^25^, anti-MX1 autoantibody–positive non-IPF patients were found to have significantly better prognosis than autoantibody-negative non-IPF patients (Fig. 5).

## Discussion

In this study, we identified IPF, INSIP, aPAP, and sarcoidosis-specific autoantibodies, with no overlap across diseases, by screening more than 8,000 autoantibodies. Subsequent bioinformatic analysis revealed several enriched KEGG pathways associated with each of the four diseases, again with no overlap. Such a method has never been used for the study of inflammatory lung diseases, and our results provide the first demonstration that IPF and INSIP are serologically distinct diseases^6^,^7^,^8^,^9^. The autoantibodies identified by our comprehensive, non-biased method also provide unexpected and novel insights into the molecular mechanisms underlying pathogenesis. Autoantibody against RNA polymerase, a clinical marker of systemic sclerosis, was identified as an enriched KEGG pathway in IPF^26^. In addition, some patients with INSIP had high levels of serum autoantibodies against lysyl oxidase–like 2, which promote cross-linking of fibrillar collagen I in fibrotic tissue^27^.

In this study, we measured IgA and IgM as well as the IgG class of anti-MX1 autoantibody to increase diagnostic sensitivity, as illustrated by the anti-cardiolipin autoantibody in antiphospholipid syndrome^28^. The autoantibody isotype profile is associated with the specific clinical features of some autoimmune diseases. For example, IgM-rheumatoid factor (RF) is primarily detected in patients with rheumatoid arthritis, whereas IgA-RF is predominantly associated with extra-articular manifestations^29^. In this study, the number of patients with each isotype of anti-MX1 autoantibody was relatively small; therefore, their clinical characteristics should be explored in the future larger-scale studies.

To eliminate selection bias in our cross-sectional study, we enrolled all consecutive patients with clinically diagnosed chronic fibrosing IIPs who visited our hospital during a certain period. The frequency of anti-ARS autoantibody positivity in our patients with IIPs was 11.4%, consistent with previous reports^16^. Although our study was limited by a relatively small study cohort consisting exclusively of patients of Japanese nationality who visited a referral hospital in our district, we speculate that 20–35% of general population of chronic fibrosing IIPs are positive for anti-MX1 or anti-ARS autoantibody. We found that anti-MX1 autoantibody–positive patients with IIPs were predominantly female, and that their morphological characteristics observed via HRCT were not in accordance with the usual interstitial pneumonia pattern^10^, but were consistent with some of the CT findings characteristic of INSIP^30^. We also found that anti-MX1 autoantibody–positive patients with IIPs had relatively good prognoses. If the clinical characteristics of both anti-MX1 autoantibody and anti-ARS autoantibody–positive patients with chronic fibrosing IIPs, including response to the recently developed drugs for IPF^31^,^32^, could be clarified in more detail, we would be able to avoid the risk of surgical lung biopsy in a substantial number of non-IPF patients^33^; at present, biopsy is required for differential diagnosis of the patients belonging to the non-IPF category according to current ATS/ERS criteria. In our study, positivity of anti-MX1 autoantibody was not significantly associated with the category of IPAF^13^. It was important that anti-MX1 autoantibody was also detected among non-IPAF patients with IIPs who were less associated with known autoimmune backgrounds. We believe that anti-MX1 autoantibody have the potential to expand the definition of autoimmunity in IIPs.

The role of anti-MX1 autoantibody in the molecular pathogenesis of interstitial pneumonia remains unclear. Indeed, the roles of all autoantibodies associated with interstitial pneumonia, including anti-MDA5 and anti-ARS, in the pathogenesis of interstitial pneumonias remain to be elucidated. However, they are thought to mediate disease pathogenesis, rather than reflecting secondary consequences of tissue damage^34^. Patients with autoantibodies to different ARSs, which have analogous functions, exhibit similar clinical symptoms, despite the fact that each ARS molecule is immunologically distinct. Although eight anti-ARS autoantibodies have been reported, cases in which two or more anti-ARS autoantibodies co-exist are very rare^15^,^16^. In our study, anti-MX1 and anti-ARS autoantibodies were mutually exclusive in patients with IIPs. Therefore, we believe that the anti-MX1 autoantibody is involved in the pathogenesis of interstitial pneumonia in IIP patients positive for this antibody.

Viral infections and type-I interferon signaling responses are involved in the pathogenesis of interstitial pneumonia^35^. In IPF, local virus antigens in lung tissues or serum antibodies against viruses are highly prevalent^36^,^37^. Originally, NSIP was the pathological term for describing the interstitial pneumonia associated with HIV infection^38^. Results from a genome-wide scan in Finnish familial interstitial pneumonia identified ELMO domain–containing 2, which regulates antiviral responses through type I interferon signaling, as a candidate gene for IPF^39^. Furthermore, among patients with clinically amyopathic dermatomyositis, an antibody against MDA5, a type I interferon–related cytosolic viral RNA sensor, is associated with rapidly progressing interstitial pneumonia^14^. Like MDA5, MX1 is also a type I interferon–inducible antiviral protein. However, the induction of these antiviral proteins by chronic exposure to viral infection does not always lead to the production of autoantibodies against them^40^,^41^. In cohort 2, only one of four patients positive for anti–hepatitis C virus antibody and none of 12 patients positive for anti–hepatitis B core antibody were positive for anti-MX1 autoantibody. In the protein array analysis, autoantibody against IFI44L, another interferon-inducible protein, was identified as IPF-specific, but there was no correlation between the titers of anti-MX1 and anti-IFI44L. Moreover, the degree of MX1 expression in surgical lung biopsy specimens did not differ significantly between patients with IPF and INSIP, despite the preferential increase in anti-MX1 autoantibody levels in patients with INSIP. Therefore, although elevated production of MX1 protein by hyperplasic airway epithelium and its relationship with local type I interferon and plasmacytoid dendritic cells remain interesting themes in interstitial pneumonia, our current hypothesis is that viral infection, which is followed by elevated expression of MX1, is not directly related to anti-MX1 autoantibody production. Studies of the function of autoantibodies against type I interferon–related antiviral proteins, including MX1 and MDA5, in interstitial pneumonia might reveal a pathogenic relationship between viral infections, type I interferon signaling, and autoimmunity.

Although today’s ATS/ERS criteria are a widely applied standard for categorizing IIPs according to radiological and/or pathological morphologies, the safety of surgical lung biopsy has long been a matter of debate^33^. Autoantibodies, including anti-ARS and the anti-MX1 autoantibody identified here, will help facilitate the clinical diagnosis of IIPs and identify more homogeneous subgroups within patients with IIPs, thereby promoting molecular pathogenetic studies, including genome studies, specifically targeted at well-defined patient populations.

## Methods

### Patients and sera

This study involved three independent cohorts of patients with chronic fibrosing IIPs. No patients were enrolled in multiple cohorts (Fig. 1).

For the protein array study, 10 patients with IPF and INSIP (cohort 1, Fig. 1A) were registered. All received surgical lung biopsy and satisfied MDD at National Hospital Organization Kinki-Chuo Chest Medical Center^1^,^2^,^10^. Prior to sample collection for the protein array experiment, all patients were followed up for an average of 55 months (range, 31–81) in the IPF group and 121 months (range, 59–135) in the INSIP group. Two patients in the INSIP group suggestive of CVDs but not fulfilling the American College of Rheumatology criteria during the follow-up period were excluded from the study. Ten patients with aPAP and sarcoidosis and 10 healthy controls were also included in the study (Fig. 1A). Diagnosis of aPAP was performed as described previously^42^. Sarcoidosis was diagnosed when clinical and radiological findings were supported by histological evidence of non-caseating epithelioid cell granulomas, after exclusion of other known causes of granulomatosis. Their clinical characteristics are presented in Table 1.

For the cross-sectional study, we enrolled 114 consecutive patients, diagnosed with chronic fibrosing IIPs based on ATS/ERS criteria, who visited the outpatient office of Osaka University Hospital between February 2014 and October 2014 (cohort 2). Serum anti-ARS autoantibody was measured by ELISA, as reported previously^16^. Pulmonary function data are presented as percentages of predicted values. High-resolution computed tomography (HRCT) images were obtained using various 64- or 320-channel multidetector CT scanners with a clinical radiation dose and reconstructed with 0.5- or 0.625-mm section thickness. The images were independently reviewed by three thoracic radiologists (MY, KU, and OH) who were blinded to the patients’ clinical information. HRCT diagnosis was performed according to the criteria in the international IPF guidelines^10^. CT findings were evaluated as described previously^30^. Disagreements with respect to distribution, diagnosis, and findings were resolved by majority. The positivity of anti-nuclear antibody (ANA) was defined by any titer with a nucleolar or centromere staining pattern, or by a titer of at least 1:320 with a diffuse, speckled or homogeneous staining pattern^13^. The positivity of rheumatoid factor (RF) was defined by a titer of twice the upper limit of normal or more^13^.

The nested case–control study used a retrospective cohort of 155 patients with chronic fibrosing IIPs registered between 2005 and 2009 at National Hospital Organization Kinki-Chuo Chest Medical Center (cohort 3). Modified ILD-GAP index was calculated from data at registration, and patients were classified into three stages, stage I: index score 0–3, stage II: index score 4–5, and stage III: index score 6–8^24^,^25^.

In both cohorts 2 and 3, patients were classified as having IPF based on the 2011 ATS/ERS/JRS/ALAT^10^ criteria and the remaining patients were classified as non-IPF (Fig. 1B,C). In total, 40 healthy volunteers were recruited. All sera and clinical data were collected after the subjects provided written informed consent in accordance with the approved institutional review board protocols of each institution. All experiments were approved by the ethics committee of Osaka University and Kinki-Chuo Chest Medical Center, and conducted according to its guidelines.

### Protein arrays

ProtoArray Human Protein Microarrays v5.0 (Invitrogen, Carlsbad, CA, USA) were purchased and used according to the manufacturer’s instructions.

After blocking for 1 h at 4 °C and washing, arrays were incubated in quadriPERM dishes (Greiner Bio-One, Frickenhausen, Germany) placed on a horizontal shaker (50 rpm) for 90 min at 4 °C with individual sera diluted at 1:500 in 5 mL washing buffer (0.1% Tween 20 [v/v], 1% BSA [w/v] in PBS). After washing, binding of IgG was detected by incubation with Alexa Fluor 647–conjugated goat anti–human IgG (Invitrogen, Waltham, MA, USA) diluted 1:2000 in assay buffer for 90 min at 4 °C. The arrays were washed again and dried by centrifugation. Arrays were scanned at a 10-μm resolution on a microarray scanner (Axon 4200AL with GenePix Pro Software; Molecular Devices, Sunnyvale, CA, USA), and fluorescence was detected according to the manufacturer’s instructions. Images were saved as 16-bit TIFF files, and analysis was performed using GenePix. The median net intensity in relative fluorescence units is reported for each spot.

### Identification of disease-specific immunogenic proteins

Immunogenic proteins were identified as described by Gnjatic *et al*.^18^,^19^. For each antigen (*i*), using normalized values across all arrays (healthy controls plus the four disease cohorts), the lower and upper quartile values [*l(i)* and *u(i)*, respectively] and the interquartile differences [IQR(*i*) = *u*(*i*) − *l*(*i*)] were calculated. To define a positive signal (*S*) for each antigen, a cutoff (*C*) value of 2.5 × IQR(*i*) was determined. Samples with *S* > *C* were selected, and the signal-to-cutoff ratio (*S*/*C*) was estimated for each sample. For each cohort, the selected *S* > *C* samples were labeled as responders and counted. Where *N*_*res*_ is the number of responders thus defined, *N* is the total number of samples for the cohort, and *S*_*i*_ is the signal for each responder (and control), the average intensity (*I*_*cohort*_) was estimated as:


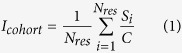


and the frequency of the responders (*F*_*cohort*_) was estimated as:


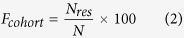


For each immunogenic protein, the antigenic score (*AS*) was estimated as:


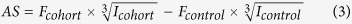


Antigens were judged to have displayed significant immunogenicity within each cohort, which was attributable to the corresponding disease, if *AS* was greater than 30. The immunogenic proteins within each cohort were ranked by *AS* and further examined to identify cohort-specific antigen groups. *AS* thresholds were incrementally increased to specifically identify antigens that exhibited substantially higher responses in a particular disease cohort than in the other cohorts.

### Functional analysis by characterization of enriched biological associations

Kyoto Encyclopedia of Genes and Genomes (KEGG) pathway data were used to assign functional annotations to the cohort-specific antigen groups. The enrichment of specific KEGG-pathway associations within each cohort was estimated by Fisher’s exact test within TargetMine^43^. To control the false discovery rate, the inferred *p* values were further adjusted for multiple comparisons using the Benjamini and Hochberg procedure, and pathway annotations were considered significant if the adjusted *p* value was ≤0.05.

### Immunoprecipitation

Cell lysates from HEK293 cells transfected with pFLAG-CMV-4 expression vector (Sigma-Aldrich, St. Louis, MO, USA) encoding the full-length cDNA for human MX1 were immunoprecipitated using sera from representative patients and protein G–Sepharose beads (GE Healthcare, Buckinghamshire, UK). The precipitates were electrophoresed and detected with a horseradish peroxidase–conjugated anti-FLAG antibody (Sigma-Aldrich).

### Immunohistochemistry

Tissue sections were deparaffinized, pretreated with microwave radiation, blocked with 2% normal goat serum, and incubated with a primary anti–human MX1 antibody (1:425, rabbit polyclonal; Sigma-Aldrich). For immunodetection, the ABC technique (VECTASTAIN Elite Kit; Serva, Heidelberg, Germany) was used according to the manufacturer’s instructions. Visualization of peroxidase localization was performed using 3,3-diaminobenzidine as a substrate.

### Preparation of recombinant His-tagged MX1 protein

Full-length cDNA of human MX1 (GenBank Accession Number: NM_002462) was subcloned into the *Bam*HI–*Xho*I sites of pET28a (+) (Novagen, Madison, WI, USA) to add a 3′ His-tag sequence. Using this subcloned plasmid as a template, the MX1 gene (including the His-tag sequence) was amplified by PCR and inserted between the *Bam*HI and *Hind*III sites of the pFastBac1 vector (Invitrogen) for baculovirus expression. Sequence analysis was performed to confirm that the construct contained the correct full-length MX1 gene and 3′ His tag. The expression plasmid was transformed into *E. coli* DH10Bac (Invitrogen) to generate bacmid DNA containing the MX1 gene. For baculovirus production, the bacmid DNA was transfected into SF-9 cells using the Cellfectin reagent (Invitrogen), and a baculovirus stock was prepared from the culture supernatant. Hi-5 cells were infected with baculovirus and incubated for 72 h at 27 °C. After incubation, the cells were harvested by centrifugation and lysed by passaging through a 20-G needle. Soluble cell lysate containing recombinant MX1 protein was separated by centrifugation and applied onto a Ni Sepharose 6 Fast Flow Resin Column (GE Healthcare). The column was washed with PBS containing 25 mM imidazole, and purified His-tagged MX1 protein was eluted with PBS containing 100 mM imidazole.

### ELISA

For detection of anti-MX1 autoantibodies, 100 μL/well of recombinant MX1 antigen solution (8 μg/mL in PBS) was added to 96-well microtiter plates (MaxiSorp; Nunc, Rochester, NY, USA) and incubated overnight at 4 °C. The plates were then washed twice with PBS and blocked overnight with PBS containing 1% bovine serum albumin (BSA) and 5% sucrose at 4 °C. Sera from patients and normal healthy donors were diluted 1:100 in phosphate buffer containing 500 mM sodium chloride, 0.15% Tween 20, 0.2% BSA, 1% casein, and 0.2 mg/mL *E. coli* extract and applied at 100 μL/well. After incubation for 60 min at 25 °C, the wells were washed four times with PBS containing 0.05% Tween 20 (PBST). To detect anti-MX1 autoantibodies of the IgG, IgA, and IgM classes, the following secondary antibodies were used: peroxidase-conjugated anti–human IgG (MBL code: 208), peroxidase-conjugated anti–human IgA (MBL code: 210), and peroxidase-conjugated anti–human IgM (MBL code: 212). These secondary antibodies were diluted 1:2000 in 20 mM HEPES, 135 mM NaCl, 1% BSA, and 0.1% p-hydroxyphenylacetic acid, and applied at 100 μL/well. After incubation for 60 min at 25 °C, the wells were washed four times with PBST, and bound antibodies were detected using 3,3′,5,5′-tetramethylbenzidine as a substrate for peroxidase. After incubation for 30 min at 25 °C, the reaction was stopped by the addition of 100 μL/well of 0.5 N sulfuric acid. Absorbance values at 450–620 nm were measured and used for data analysis. Baseline cut-off values of 0.237, 0.312, and 0.450 were used for IgG, IgA, and IgM class detection, respectively. These cut-off values were determined by calculating the mean absorbance values of healthy donors plus 6 SDs.

### Statistical analysis

Continuous and ordinal data are expressed as medians with range (minimum and maximum), and categorical data were expressed as percentages. Clinical characteristics of anti-MX1 autoantibody–positive and –negative patients with IIPs were compared by Wilcoxon rank tests for continuous data, Fisher’s exact tests for categorical data, or chi-squared test for ordinal data. The titers of IgG-, IgA-, and IgM-class anti-MX1 autoantibody were compared by the Steel–Dwass procedure, adjusting for multiple comparisons. Multivariable stepwise logistic regression was used to identify the factors associated with anti-MX1 autoantibody–positive IIPs, and odds ratios and associated 95% confidence intervals were calculated. The generalized and stratified Wilcoxon test was used to compare the prognosis of anti-MX1 autoantibody–positive and –negative patients, before and after the adjustment for the staging of modified ILD-GAP Index, respectively. Differences with *p* values less than 0.05 were considered statistically significant. Statistical analyses were carried out using the JMP Pro software, version 11.0.0 (SAS Institute, Cary, NC, USA) and STATA/IC 12.1 (StataCorp LP, Collage Station, TX, USA).

## Additional Information

**How to cite this article**: Hamano, Y. *et al*. Classification of idiopathic interstitial pneumonias using anti–myxovirus resistance-protein 1 autoantibody. *Sci. Rep.* **7**, 43201; doi: 10.1038/srep43201 (2017).

**Publisher's note:** Springer Nature remains neutral with regard to jurisdictional claims in published maps and institutional affiliations.

## Supplementary Material

Supplementary Information

## Figures and Tables

**Table 1:** Characteristics of patients whose samples were used for protein arrays (cohort 1).

Disease	Number (male:female)	Age Mean (range)	Serum marker Mean (range)
IPF	10 (9:1)	62.9 (47–71)	KL-6 1310.7 (376–2630 U/mL)
INSIP	8 (4:4)	58.5 (42–73)	KL-6 2049 (384–4770 U/mL)
aPAP	10 (4:6)	49.3 (30–68)	Anti–GM-CSF antibody 76.5 (1.8–177.0 μg/mL)
Sarcoidosis	10 (1:9)	58.9 (40–74)	ACE 20.4 (13.4–33.9 U/L)
HC	10 (8:2)	49.9 (40–59)	

Abbreviations: HC, healthy control; KL-6, Krebs von den Lungen-6; GM-CSF, granulocyte–macrophage colony stimulating factor; ACE, angiotensin-converting enzyme.

**Table 2:** List of the top 15 proteins with enhanced antigenicity specific to IPF, INSIP, aPAP, or sarcoidosis cohorts relative to background.

Gene	Symbol	*F*_*control*_	*F*_*cohort*_	*I*_*control*_	*I*_*cohort*_	*AS*
**IPF**						
NM_003406.2	*YWHAZ*	13.16	50	17.1	21.67	122.3
NM_003564.1	*TAGLN2*	15.79	30	23.65	50.45	87.21
NM_182970.2	*RIMS4*	0	40	0	9.59	84.97
NM_005423.1	*TFF2*	5.26	30	5.46	24.75	81.97
NM_022839.2	*MRPS11*	5.26	30	12.93	31.35	81.66
NM_006674.2	*HCP5*	5.26	50	5.93	5.09	80.06
NM_012425.2	*RSU1*	13.16	50	16.64	7.02	79.08
BC007565.1	*PLCG2*	5.26	30	6.08	21.68	77.57
BC068456.1	*YWHAZ*	5.26	30	6.37	19.56	74.47
NM_052849.2	*C15orf57*	5.26	40	5.96	7.93	73.81
NM_016467.1	*ORMDL1*	36.84	70	40.23	4.21	72.82
BC015932.2	*IFI44L*	5.26	60	8.56	2.47	72.59
NM_015417.2	*SPEF1*	13.16	50	18.13	5.9	72.23
NM_012280.1	*FTSJ1*	2.63	30	2.85	15.33	71.68
BC031300.1	*C21orf2*	5.26	30	6.11	15.46	68.63
**INSIP**						
BC007722.2	*GARS*	7.5	25	9.99	269.77	151.55
BC000594.2	*LOXL2*	27.5	50	35.08	33.68	126.39
NM_000318.1	*PEX2*	2.5	37.5	2.61	30.35	114.36
BC008730.2	*HK1*	5	25	6.48	72.83	97.92
NM_016533.4	*NINJ2*	2.5	62.5	3.22	3.81	94.44
NM_002462.2	*MX1*	10	62.5	14.47	4.31	87.28
NM_178152.1	*DCX*	10	75	15.03	2.48	86.43
NM_032369.1	*HVCN1*	50	62.5	57.35	11.02	81.71
BC024254.1	*ABI1*	5	37.5	6.53	12.48	80.46
NM_003910.2	*BUD31*	12.5	37.5	15.42	16	79.07
NM_013301.1	*CCDC106*	7.5	25	8.67	41.22	77.69
NM_145314.1	*UCMA*	10	37.5	12.02	13.27	76.76
BC019598.1	*ZMAT4*	7.5	62.5	9.54	2.57	76.06
NM_032323.1	*TMEM79*	2.5	25	2.82	29.22	74.18
BC028404.1	*VSTM2A*	10	37.5	22.18	15.54	71.4
**aPAP**						
NM_000758.2	*CSF2*	0	100	0	28.46	305.33
NM_032498.1	*RHOXF2*	15.79	70	18.34	7.26	117.2
NM_199168.2	*CXCL12*	10.53	50	11.23	13.34	107.35
BC024244.1	*SPSB3*	39.47	90	44.29	4.51	104.38
BC030280.1	*KIAA0513*	0	30	0	34.28	97.45
NM_002399.2	*MEIS2*	10.53	30	21.38	57.92	94.7
NM_003339.2	*UBE2D2*	7.89	30	8.87	35.82	90.02
BC034236.1	*LINC00663*	7.89	30	9.03	31.43	85.65
BC024241.2	*CDO1*	7.89	40	9.73	13.51	85.54
BC000954.1	*CBX3*	10.53	50	26.23	9.92	81.2
NM_016400.2	*HYPK*	5.26	50	6.84	5.08	79.13
BC022958.1	*TSTD2*	10.53	20	14.29	88.5	74.83
NM_005698.2	*SCAMP3*	13.16	40	17.2	12.12	74.68
NM_153649.2	*TPM3*	5.26	50	6.98	4.24	73.98
BC030597.1	*ATRIP*	5.26	60	6.57	2.41	73.87
**Sarcoidosis**						
NM_001007071.1	*RPS6KB2*	7.89	70	11.07	8.61	132.4
NM_007375.3	*TARDBP*	10.53	60	11.61	7.57	106.18
NM_003831.1	*RIOK3*	15.79	50	16.99	12.73	99.77
BC001487.2	*TARDBP*	7.89	50	9.01	8.86	94.44
NM_138484.1	*SGOL1*	5.26	60	6.97	4.36	91.05
NM_017817.1	*RAB20*	34.21	70	41.31	6.74	90.92
BC050537.2	*MFSD6*	7.89	40	8.61	13.33	86.22
NM_130897.1	*DYNLRB2*	13.16	70	23.87	3.62	83.6
BC028237.1	*GDF10*	47.37	60	59.42	13.24	82.53
BC032708.1	*TBL1X*	0	30	0	20.71	82.38
NM_016216.2	*DBR1*	10.53	50	21.52	8.6	80.93
BC018142.1	*CARD14*	5.26	50	6.87	5.38	80.76
BC054520.1	*MEF2D*	5.26	50	6.66	5.14	79.63
BC038381.1	*BCAS4*	5.26	20	6.28	74.19	77.76
NM_032926.1	*TCEAL3*	2.63	30	3.63	18.87	76.24

See complete list in Supplementary Table S1. For each disease cohort, the control group consisted of healthy controls plus patients with the other three diseases. Frequency (*F*) is the percentage of sera that reacted with a given antigen with value greater than 2.5 × the interquartile difference above the 75th percentile (i.e., the cutoff). The intensity (*I*) represents the average ratio of observed reactivity over the cutoff. The antigenic score (*AS*) was estimated as 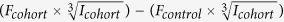. Antigens were ranked according to the *AS* scores within each disease cohort. The top 15 antigens within each disease cohort are listed in descending order of *AS*.

**Table 3:** Enriched Kyoto Encyclopedia of Genes and Genomes (KEGG) pathway associations (*p* ≤ 0.05) for proteins with enhanced antigenicity associated with IPF, INSIP, aPAP, or sarcoidosis cohorts (as listed in Supplementary Table S1).

Enriched KEGG pathway	KEGG pathway ID	*p*-value	Associated genes
**IPF**			
Epstein-Barr virus infection	hsa05169	3.59367E-05	*PLCG2, POLR2L, POLR3K, YWHAZ*
RNA polymerase	hsa03020	0.000637682	*POLR2L, POLR3K*
Cytosolic DNA-sensing pathway	hsa04623	0.002387945	*POLR2L, POLR3K*
Pyrimidine metabolism	hsa00240	0.006154429	*POLR2L, POLR3K*
Purine metabolism	hsa00230	0.015536822	*POLR2L, POLR3K*
Metabolic pathways	hsa01100	0.027910673	*PHGDH, PLCG2, POLR2L, POLR3K*
Glycine, serine and threonine metabolism	hsa00260	0.044458806	*PHGDH*
**INSIP**			
Type II diabetes mellitus	hsa04930	0.000873218	*HK1, MAPK10, PRKCZ*
Aminoacyl-tRNA biosynthesis	hsa00970	0.001926137	*GARS, MARS, QARS*
Selenocompound metabolism	hsa00450	0.002240339	*MARS, PAPSS2*
2-Oxocarboxylic acid metabolism	hsa01210	0.002240339	*ACO2, GPT2*
Biosynthesis of amino acids	hsa01230	0.002571216	*ACO2, GPT2, SHMT2*
Glyoxylate and dicarboxylate metabolism	hsa00630	0.004431551	*ACO2, SHMT2*
Insulin signaling pathway	hsa04910	0.017360641	*HK1, MAPK10, PRKCZ*
Amino sugar and nucleotide sugar metabolism	hsa00520	0.017708207	*CYB5R1, HK1*
Lysine biosynthesis	hsa00300	0.019434624	*ACO2*
Butirosin and neomycin biosynthesis	hsa00524	0.024127735	*HK1*
Influenza A	hsa05164	0.033284871	*MAPK10, MX1, RSAD2*
Cyanoamino acid metabolism	hsa00460	0.033322731	*SHMT2*
Adipocytokine signaling pathway	hsa04920	0.033887009	*MAPK10, RXRA*
Progesterone-mediated oocyte maturation	hsa04914	0.049314314	*CDK1, MAPK10*
**aPAP**			
Nicotinate and nicotinamide metabolism	hsa00760	0.007881076	*NMRK1, QPRT*
Toxoplasmosis	hsa05145	0.01706976	*IL10RB, PIK3R5, TAB1*
Cytokine-cytokine receptor interaction	hsa04060	0.027595171	*BMPR2, CSF2, CXCL12, IL10RB*
Jak-STAT signaling pathway	hsa04630	0.03365456	*CSF2, IL10RB, PIK3R5*
Shigellosis	hsa05131	0.034504413	*HCLS1, UBE2D2*
Fc epsilon RI signaling pathway	hsa04664	0.04433339	*CSF2, PIK3R5*
Influenza A	hsa05164	0.045730626	*OAS1, PIK3R5, PLG*
**Sarcoidosis**			
Oocyte meiosis	hsa04114	0.019485662	*MAD2L1, SGOL1*
PI3K–Akt signaling pathway	hsa04151	0.025741505	*PPP2R3B, RPS6K2, VWF*

**Table 4:** Comparison of clinical characteristics between anti-MX1 autoantibody–positive and –negative patients with IIPs (n = 114) in cohort 2.

Variables	Anti-MX1 autoantibody		*p* value
	Positive (n = 20)	Negative (n = 94)	
Age (years)	75 (39–86)	73 (33–86)	0.72
Female, n (%)	11 (55.0%)	24 (25.5%)	0.02*
Smoking history (pack-year)	15.75 (0–80)	28.5 (0–135)	0.33
ANA positive, n (%)	0/19 (0.0%)	10/91 (11.0%)	0.21
RF positive, n (%)	2/19 (10.5%)	17/93 (18.3%)	0.52
CK (U/L)	72 (45–157)	97 (28–976)	0.14
CRP (mg/dL)	0.12 (<0.04–1.1)	0.09 (<0.04–1.66)	0.96
KL-6 (U/mL)	580 (310–2937)	817.5 (99–4576)	0.39
SP-D (ng/mL)	220 (64.1–761)	186 (19.8–1300)	0.30
FVC (%)	84.8 (44.9–107.6)	79.9 (27.9–136.9)	0.81
D_LCO_ (%)	45.4 (20.2–88.0)	61.6 (4.2–147.5)	0.05
Use of glucocorticoid, n (%)	6 (30.0%)	22 (23.4%)	0.57
Use of pirfenidone, n (%)	1 (5.0%)	8 (8.5%)	1.00
Diagnosis of IPF, n (%)	4 (20.0%)	15 (16.0%)	0.74
**HRCT findings**			
Spared area (%)	57.5 (38.3–93.3)	65.8 (13.3–91.7)	0.24
Predominantly lower, n (%)	14 (70.0%)	85 (90.4%)	0.02*
Predominantly peripheral, n (%)	11 (55.0%)	77 (81.9%)	0.02*
Predominantly peribronchovascular, n (%)	9 (45.0%)	42 (44.7%)	1.00
Asymmetric distribution, n (%)	0 (0.0%)	3 (3.2%)	1.00
GGA with traction bronchiectasis, n (%)	19 (95.0%)	81 (86.2%)	0.46
GGA without traction bronchiectasis, n (%)	17 (85.0%)	72 (76.6%)	0.56
Air-space consolidation, n (%)	9 (45.0%)	38 (40.4%)	0.80
Honeycombing, n (%)	6 (30.0%)	34 (36.2%)	0.80
Intralobular reticular opacity, n (%)	16 (80.0%)	73 (77.7%)	1.00
Emphysema, n (%)	7 (35.0%)	35 (37.2%)	1.00
Traction bronchiectasis, n (%)	3 (15.0%)	18 (19.2%)	1.00
Sub pleural sparing, n (%)	0 (0.0%)	4 (4.3%)	1.00
Upper lobe subpleural line, n (%)	1 (5.0%)	0 (0.0%)	0.18

Abbreviations: ANA, anti-nuclear antibody; RF, rheumatoid factor; CK, creatine kinase; CRP, C reactive protein; KL-6, Krebs von den Lungen-6; SP-D, surfactant protein D; FVC, forced vital capacity; D_LCO_, carbon monoxide diffusing capacity; GGA, ground glass attenuation. *p < 0.05.

**Table 5:** Stepwise multivariable logistic regression analysis for the characterization of anti-MX1 autoantibody–positive IIPs in cohort 2.

Variables	OR (95% CI)	*p* value
Female	1.73 (1.02–2.94)	0.04*
D_LCO_ (%) per 10% increment	0.80 (0.63–1.02)^†^	0.07
Predominantly lower & peripheral distribution	0.58 (0.34–0.98)	0.04*

Abbreviations: OR, odds ratio; CI, confidence interval; D_LCO_, carbon monoxide diffusing capacity. *p < 0.05. ^†^For every 10% increment in D_LCO_.
