# Supplementary material for: Classification of idiopathic interstitial pneumonias using anti–myxovirus resistance-protein 1 autoantibody
Source: Sci Rep. 2017 Feb 23;7:43201. doi: 10.1038/srep43201 (PMC5322336; doi:10.1038/srep43201)

Supplementary Information

Classification of idiopathic interstitial pneumonias using anti–myxovirus resistance-protein 1 autoantibody

Yoshimasa Hamano[1], Hiroshi Kida[1,10], Shoichi Ihara[1], Akihiro Murakami[2], Masahiro Yanagawa[3], Ken Ueda[3], Osamu Honda[3], Lokesh P. Tripathi[4], Toru Arai[5], Masaki Hirose[5], Toshimitsu Hamasaki[6], Yukihiro Yano[7], Tetsuya Kimura[9], Yasuhiro Kato[1,9,10], Hyota Takamatsu[1,9,10], Tomoyuki Otsuka[1,10], Toshiyuki Minami[1,10], Haruhiko Hirata[1,10], Koji Inoue[1,10], Izumi Nagatomo[1,10], Yoshito Takeda[1,10], Masahide Mori[7], Hiroyoshi Nishikawa[8], Kenji Mizuguchi[4], Takashi Kijima[1,10], Masanori Kitaichi[5], Noriyuki Tomiyama[3], Yoshikazu Inoue[5], and Atsushi Kumanogoh[1,9,10]

[1]Department of Respiratory Medicine, Allergy and Rheumatic Diseases, Osaka University Graduate School of Medicine, 2-2 Yamadaoka, Suita City, Osaka 565-0871, Japan; [2]Medical & Biological Laboratories Co., Ltd., Ina Laboratory, 1063-103 Terasawaoka, Ina City, Nagano 396-0002, Japan; [3]Department of Radiology, Osaka

University Graduate School of Medicine, 2-2 Yamadaoka, Suita City, Osaka 565-0871, Japan; [4]National Institutes of Biomedical Innovation, Health and Nutrition, 7-6-8 Saitoasagi, Ibaraki City, Osaka 567-0085, Japan; [5]National Hospital Organization Kinki-Chuo Chest Medical Center, 1180 Nagasone-Cho, Kita-Ku, Sakai City, Osaka 591-8555, Japan; [6]Office of Biostatistics and Data Management, National Cerebral and Cardiovascular Center, 5-7-1 Fujishirodai, Suita City, Osaka 565-8565, Japan; [7]National Hospital Organization Toneyama National Hospital, 5-1-1 Toneyama, Toyonaka City, Osaka 560-8552, Japan; [8]Department of Experimental Immunology, WPI Immunology Frontier Research Center, Osaka University, Yamadaoka 3-1, Suita City, Osaka 565-0871 Japan; [9]Department of Immunopathology, WPI Immunology Frontier Research Center, Osaka University, Yamadaoka 3-1, Suita City, Osaka 565-0871, Japan; and [10]AMED, CREST, Suita City, Osaka 565-0871, Japan

Corresponding Author

Hiroshi Kida, MD, PhD

Address: 2-2 Yamadaoka, Suita City, Osaka 565-0871, Japan

Phone: 81-66879-3833

Fax: 81-66879-3839

E-mail: hiroshi.kida@imed3.med.osaka-u.ac.jp

Supplementary Table S1: Complete list of proteins with enhanced antigenicity specific to IPF, INSIP, aPAP, or sarcoidosis cohorts

IPF						
**Gene**	**Symbol**	$F_{control}$	F_{cohort}	$I_{control}$	I_{cohort}	AS
NM_003406.2	*YWHAZ*	13.16	50	17.1	21.67	122.3
NM_003564.1	*TAGLN2*	15.79	30	23.65	50.45	87.21
NM_182970.2	*RIMS4*	0	40	0	9.59	84.97
NM_005423.1	*TFF2*	5.26	30	5.46	24.75	81.97
NM_022839.2	*MRPS11*	5.26	30	12.93	31.35	81.66
NM_006674.2	*HCP5*	5.26	50	5.93	5.09	80.06
NM_012425.2	*RSU1*	13.16	50	16.64	7.02	79.08
BC007565.1	*PLCG2*	5.26	30	6.08	21.68	77.57
BC068456.1	*YWHAZ*	5.26	30	6.37	19.56	74.47
NM_052849.2	*C15orf57*	5.26	40	5.96	7.93	73.81
NM_016467.1	*ORMDL1*	36.84	70	40.23	4.21	72.82
BC015932.2	*IFI44L*	5.26	60	8.56	2.47	72.59
NM_015417.2	*SPEF1*	13.16	50	18.13	5.9	72.23
NM_012280.1	*FTSJ1*	2.63	30	2.85	15.33	71.68
BC031300.1	*C21orf2*	5.26	30	6.11	15.46	68.63
NM_001290.1	*LDB2*	2.63	50	3.22	2.9	68.09
BC040107.1	*CRCP*	18.42	40	30.02	14.7	67.97
BC015329.1	*TAGLN3*	15.79	20	18.07	78.13	67.43
NM_016310.2	*POLR3K*	13.16	30	17.76	22.23	66.6
NM_006623.1	*PHGDH*	0	10	0	290.73	66.25
NM_021128.3	*POLR2L*	5.26	20	7.87	50.25	65.94
NM_144659.1	*TCP10L*	10.53	50	11.43	3.58	65.06
NM_023940.1	*RASL11B*	13.16	50	22.5	5.35	64.96
BC002559.1	*YTHDF2*	13.16	50	19.03	4.54	63.77
BC009462.1	*METTL21A*	5.26	60	5.52	1.51	63.29
NM_020961.2	*METTL14*	10.53	50	11.19	3.26	62.97

NM_025125.2	*TMEM254*	2.63	60	3.44	1.33	62.47
BC059374.1	*STK31*	13.16	50	22.93	4.95	62.28
BC025399.1	*PROK1*	23.68	60	25.69	3.15	62.27
NM_012191.1	*NAT6*	10.53	40	11.66	6.3	62.2
NM_023930.1	*KCTD14*	7.89	50	8.86	2.85	62.04
NM_021824.2	*NIF3L1*	10.53	30	10.99	14.2	61.67
NM_001261.2	*CDK9*	15.79	30	19.77	19.27	60.66
NM_080416.1	*SEPT4*	18.42	30	39	36.62	60.63
BC012341.1	*TIMMDC1*	18.42	30	22.98	21.59	60.56
BC016979.1	*NECAB2*	10.53	50	13.25	3.16	60.13
BC060768.1	*ZNF449*	2.63	40	3.52	3.98	59.87
BC016911.1	*RECQL5*	13.16	50	15.57	3.36	59.34
BC011454.1	*AMOTL2*	5.26	50	6	2.16	58.64
BC012901.1	*ROGDI*	7.89	40	8.88	4.73	58.27
NM_003168.1	*SUPT4H1*	7.89	40	11.09	4.99	57.28
**INSIP**						
**Gene**	**Symbol**	$F_{control}$	F_{cohort}	$I_{control}$	I_{cohort}	AS
BC007722.2	*GARS*	7.5	25	9.99	269.77	151.55
BC000594.2	*LOXL2*	27.5	50	35.08	33.68	126.39
NM_000318.1	*PEX2*	2.5	37.5	2.61	30.35	114.36
BC008730.2	*HK1*	5	25	6.48	72.83	97.92
NM_016533.4	*NINJ2*	2.5	62.5	3.22	3.81	94.44
NM_002462.2	*MX1*	10	62.5	14.47	4.31	87.28
NM_178152.1	*DCX*	10	75	15.03	2.48	86.43
NM_032369.1	*HVCN1*	50	62.5	57.35	11.02	81.71
BC024254.1	*ABI1*	5	37.5	6.53	12.48	80.46
NM_003910.2	*BUD31*	12.5	37.5	15.42	16	79.07
NM_013301.1	*CCDC106*	7.5	25	8.67	41.22	77.69
NM_145314.1	*UCMA*	10	37.5	12.02	13.27	76.76
BC019598.1	*ZMAT4*	7.5	62.5	9.54	2.57	76.06
NM_032323.1	*TMEM79*	2.5	25	2.82	29.22	74.18
BC028404.1	*VSTM2A*	10	37.5	22.18	15.54	71.4

NM_001814.1	*CTSC*	0	25	0	22.29	70.36
NM_005051.1	*QARS*	5	37.5	9.23	9.37	69.83
BC027729.1	*TPRXL*	15	50	17.16	5.14	69.13
BC007067.1	*NSL1*	42.5	87.5	47.58	2.34	68.65
BC103812.1	*ALKBH3*	22.5	75	25.07	1.94	68.51
NM_001098.2	*ACO2*	10	50	11.49	3.98	67.74
NM_004987.3	*LIMS1*	25	87.5	27.3	1.26	67.3
NM_018393.2	*TCP11L1*	5	37.5	6.23	7.5	67.16
BC010467.1		10	62.5	13.79	2.16	66.98
NM_001786.2	*CDK1*	10	62.5	12.46	2.02	66.59
BC024919.1	*NUBPL*	12.5	50	15.19	4.29	66.02
BC017046.1	*ANXA6*	0	25	0	17.87	65.36
NM_020239.2	*CDC42SE1*	5	62.5	5.23	1.44	65.33
NM_138501.3	*TECR*	30	87.5	34.32	1.47	65.06
NM_022823.1	*FNDC4*	15	50	19.74	4.86	64.93
BC009415.1	*KIF26A*	7.5	62.5	9.7	1.7	64.86
NM_002753.2	*MAPK10*	10	25	11.39	27.55	64.11
BC008058.1	*PRKCZ*	17.5	37.5	34.66	18.21	63.99
BC000178.2	*KCMF1*	7.5	25	10.29	25.91	63.68
BC032866.2	*EIF5*	5	50	6.86	2.81	63.66
NM_178151.1	*DCX*	5	37.5	8.11	6.76	62.81
NM_001001711.1	*DDI1*	12.5	37.5	17.42	9.66	62.44
BC052966.1	*PARG*	2.5	50	2.94	2.22	62.31
BC036767.1	*RIBC1*	10	62.5	13.39	1.76	62.12
NM_018222.2	*PARVA*	5	50	5.64	2.49	62.09
NM_002957.3	*RXRA*	7.5	37.5	8.89	6.63	61.57
BC017969.1	*RSAD2*	12.5	50	21.4	4.5	61.18
NM_004990.2	*MARS*	10	25	14.87	27.54	60.63
BC018732.1	*CYB5R1*	7.5	37.5	8.33	6.01	59.84
BC017589.2	*TPD52L3*	20	75	22.85	1.34	59.84
BC016470.2	*EME1*	12.5	50	19.71	4.01	59.72
NM_198517.2	*TBC1D10C*	10	62.5	10.97	1.44	59.65

NM_024805.1	*RBFA*	17.5	75	20.7	1.21	59.27
NM_005412.2	*SHMT2*	7.5	37.5	13.05	7.1	59.04
NM_133443.1	*GPT2*	5	50	5.35	2.08	58.45
NM_013233.1	*STK39*	17.5	75	20.28	1.15	58.37
NM_152340.1		2.5	37.5	2.62	4.09	57.34
NM_020236.2	*MRPL1*	7.5	37.5	8.76	5.48	57.33
BC009894.2	*PAPSS2*	7.5	50	10.92	2.53	57.19

**aPAP**						
**Gene**	**Symbol**	$F_{control}$	F_{cohort}	$I_{control}$	I_{cohort}	***AS***
NM_000758.2	*CSF2*	0	100	0	28.46	305.33
NM_032498.1	*RHOXF2*	15.79	70	18.34	7.26	117.2
NM_199168.2	*CXCL12*	10.53	50	11.23	13.34	107.35
BC024244.1	*SPSB3*	39.47	90	44.29	4.51	104.38
BC030280.1	*KIAA0513*	0	30	0	34.28	97.45
NM_002399.2	*MEIS2*	10.53	30	21.38	57.92	94.7
NM_003339.2	*UBE2D2*	7.89	30	8.87	35.82	90.02
BC034236.1	*LINC00663*	7.89	30	9.03	31.43	85.65
BC024241.2	*CDO1*	7.89	40	9.73	13.51	85.54
BC000954.1	*CBX3*	10.53	50	26.23	9.92	81.2
NM_016400.2	*HYPK*	5.26	50	6.84	5.08	79.13
BC022958.1	*TSTD2*	10.53	20	14.29	88.5	74.83
NM_005698.2	*SCAMP3*	13.16	40	17.2	12.12	74.68
NM_153649.2	*TPM3*	5.26	50	6.98	4.24	73.98
BC030597.1	*ATRIP*	5.26	60	6.57	2.41	73.87
NM_006116.2	*TAB1*	15.79	30	19.23	29.49	73.46
BC007340.1	*BYSL*	15.79	30	18.87	28.21	72.46
NM_013313.3	*YPEL1*	7.89	50	12.16	4.75	71.91
BC022251.1	*ZFAND1*	5.26	50	6.02	3.71	71.4
BC058886.1	*AAGAB*	2.63	30	3.06	14.53	70.15
NM_017881.1	*NMRK1*	10.53	10	13.79	588.4	70.01
NM_004227.3	*CYTH3*	7.89	40	13.3	8.92	69.66
BC025254.1	*INTS3*	10.53	40	13.45	8.83	69.23

BC028212.1	*PIK3R5*	26.32	80	29.74	1.83	68.06
NM_032316.1	*NICN1*	34.21	60	40.8	5.91	67.7
NM_012215.1	*MGEA5*	2.63	50	2.84	2.74	67.12
NM_015971.2	*MRPS7*	10.53	50	14.31	4.22	66.47
BC017570.1	*C9orf78*	5.26	40	5.64	5.81	66.26
NM_001839.1	*CNN3*	7.89	60	10.75	2.09	65.99
NM_198395.1	*G3BP1*	15.79	50	21.1	5.25	65.8
NM_024114.1	*TRIM48*	13.16	70	14.45	1.45	64.8
NM_004929.2	*CALB1*	2.63	20	3.51	39.67	64.69
NM_016816.1	*OAS1*	5.26	50	8.28	3.1	64.62
BC017059.1	*IFI16*	5.26	20	9.47	50.45	64.43
BC021551.1	*NFATC2IP*	10.53	40	12.09	6.66	63.15
BC036511.1	*MEIS1*	10.53	20	11.5	50.77	62.55
BC031695.1	*DPCD*	2.63	20	8.63	44.7	62.35
XM_293034.2		5.26	30	7.73	12.51	61.92
NM_002710.1	*PPP1CC*	10.53	50	12.5	3.24	61.51
BC060513.1	*PLG*	7.89	30	9.8	13.38	61.43
BC010033.1	*QPRT*	10.53	40	13.21	6.41	61.1
NM_032284.1	*PRPF38A*	5.26	50	5.33	2.29	60.56
NM_012145.2	*DTYMK*	7.89	40	13.13	6.18	60.27
BC005893.1	*NPPA*	15.79	60	17.65	2.18	60.11
NM_003338.2	*UBE2D2*	0	10	0	216.1	60.01
NM_000628.3	*IL10RB*	5.26	50	6.55	2.31	59.57
BC033035.1	*FLJ25758*	5.26	30	5.82	10.35	59.55
NM_052937.1	*PCMTD1*	26.32	50	30.41	5.81	59.46
NM_017907.1	*LAMTOR1*	23.68	60	27.29	2.9	58.24
BC007363.1	*BCKDK*	18.42	40	24.15	8.71	58.15
BC002769.1	*RTFDC1*	7.89	20	13.75	45.85	57.84
BC002660.1	*TMOD1*	13.16	40	18.49	6.94	57.82
NM_005335.3	*HCLS1*	7.89	40	10.68	5	57.71
NM_018452.1	*TMEM242*	5.26	50	6.13	2.05	57.43
NM_001204.3	*BMPR2*	47.37	90	52.4	1.82	57.41

NM_173796.2		7.89	30	10	11.33	57.38
NM_138818.1	*PRUNE2*	7.89	50	8.78	2.31	57.34
NM_000366.5	*TPM1*	13.16	40	16.13	6.17	57.24
NM_006755.1	*TALDO1*	10.53	50	16.34	3.18	57.21
**Sarcoidosis**						
**Gene**	**Symbol**	$F_{control}$	F_{cohort}	$I_{control}$	I_{cohort}	***AS***
NM_001007071.1	*RPS6KB2*	7.89	70	11.07	8.61	132.4
NM_007375.3	*TARDBP*	10.53	60	11.61	7.57	106.18
NM_003831.1	*RIOK3*	15.79	50	16.99	12.73	99.77
BC001487.2	*TARDBP*	7.89	50	9.01	8.86	94.44
NM_138484.1	*SGOL1*	5.26	60	6.97	4.36	91.05
NM_017817.1	*RAB20*	34.21	70	41.31	6.74	90.92
BC050537.2	*MFSD6*	7.89	40	8.61	13.33	86.22
NM_130897.1	*DYNLRB2*	13.16	70	23.87	3.62	83.6
BC028237.1	*GDF10*	47.37	60	59.42	13.24	82.53
BC032708.1	*TBL1X*	0	30	0	20.71	82.38
NM_016216.2	*DBR1*	10.53	50	21.52	8.6	80.93
BC018142.1	*CARD14*	5.26	50	6.87	5.38	80.76
BC054520.1	*MEF2D*	5.26	50	6.66	5.14	79.63
BC038381.1	*BCAS4*	5.26	20	6.28	74.19	77.76
NM_032926.1	*TCEAL3*	2.63	30	3.63	18.87	76.24
NM_178496.2	*MB21D2*	21.05	30	26.35	39.19	75.55
BC030767.1	*C5orf58*	13.16	30	18.75	30.6	75.08
NM_003820.2	*TNFRSF14*	2.63	20	2.63	54.02	72.97
BC054022.1	*PAGE2*	7.89	20	9.79	64.47	70.41
NM_199326.1	*PPP2R3B*	15.79	40	19.06	11.14	70.27
BC022258.1	*VWF*	21.05	50	22.94	6.41	69.97
BC000870.1	*TIPIN*	7.89	40	8.62	7.4	69.34
BC057783.1	*SRSF8*	7.89	60	8.54	2.16	69.05
NM_018679.2	*TCP11*	2.63	40	2.76	5.58	68.2
BC008656.1	*SPATA7*	7.89	30	11.38	18.27	67.64
NM_176869.1	*PPA2*	7.89	60	8.88	1.99	66.58

NM_017851.4	*PARP16*	10.53	20	11.71	57.82	65.63
BC016857.1	*FTH1*	13.16	50	16.55	4.42	65.53
NM_058173.1	*MUCL1*	15.79	40	24.43	11.12	64.85
XM_211837.1		10.53	50	11.54	3.47	64.14
BC013173.1	*RSPRY1*	21.05	70	22.03	1.82	63.43
BC006106.1	*RPS6KB2*	7.89	50	13.26	3.61	63.43
BC050387.1	*ANKS3*	13.16	40	16.63	7.95	63.19
NM_021026.1	*RFPL1*	2.63	30	2.8	10.63	63.16
BC040949.1	*MEF2D*	7.89	40	9.69	6.03	63.11
NM_001012979.1	*TCEAL5*	5.26	30	5.9	11.88	62.55
NM_012443.2	*SPAG6*	21.05	70	24.39	1.91	62.41
NM_022912.1	*REEP1*	7.89	30	12.21	15.23	62.15
BC002794.1	*TNFRSF14*	0	20	0	29.3	61.66
NM_014012.2	*REM1*	10.53	60	13.31	1.9	61.06
NM_004632.2	*DAP3*	2.63	20	2.64	32.28	61.04
NM_176791.2	*GTSF1L*	7.89	50	12.76	3.2	60.89
NM_007233.1	*TP53TG1*	5.26	40	5.62	4.37	59.79
NM_002395.2	*ME1*	10.53	40	18.47	7.31	59.17
BC033794.1	*HDDC3*	10.53	40	15.21	6.41	59.08
NM_001157.2	*ANXA11*	10.53	40	37.79	13.98	58.58
BC027900.1	*RHBDD1*	23.68	60	27.14	2.84	57.88
NM_002358.2	*MAD2L1*	10.53	40	12.76	5.49	57.79
BC002950.1	*C18orf8*	18.42	40	23.58	8.24	57.21

For each disease cohort, the control group included healthy controls and patients with the other three diseases. Frequency (F) is the percentage of sera that reacted to a given antigen with a value greater than 2.5× the interquartile difference above the 75th percentile (i.e., the cutoff). Intensity (I) represents the average ratio of the observed reactivity above the cutoff. The antigenic score (AS) was calculated as ($F_{cohort} \times \sqrt[3]{I_{cohort}}$) − ($F_{control} \times \sqrt[3]{I_{cohort}}$). Antigens are

ranked according to their *AS* values within each disease cohort. Disease cohort–specific antigens (AS ≥ 57.1) are listed in descending order of *AS*.

Supplementary Table S2: Comparison of clinical characteristics at diagnosis between anti-MX1 autoantibody–positive and –negative non-IPF patients in cohort 3

	**anti-MX1 antibody**		
**Variables**	**positive (n = 20)**	**negative (n = 59)**	***p* value**
Age (years)	72 (57–87)	69 (35–87)	0.52
Female, n (%)	8 (40.0%)	20 (33.9%)	0.79
KL-6 (U/mL)	948.5 (147–3450)	838.0 (151–4630)	0.90
SP-D (ng/mL)	125.5 (40.2–421)	194.5 (17.2–2660)	0.12
FVC (%)	72.7 (33.9–126.1)	78.7 (30.1–138.9)	0.26
D_{LCO} (%)	50.3 (24.1–76.0)	51.6 (6.6–116.3)	0.59
ILD-GAP staging	2 (1–3)	1 (1–3)	0.37

Abbreviations: KL-6, Krebs von den Lungen-6; SP-D, surfactant protein D; FVC, forced vital capacity; D_{LCO}, carbon monoxide diffusing capacity; ILD, interstitial lung disease.

Supplementary Figure S1: Uncropped membrane blots corresponding to Fig. 2B in the manuscript

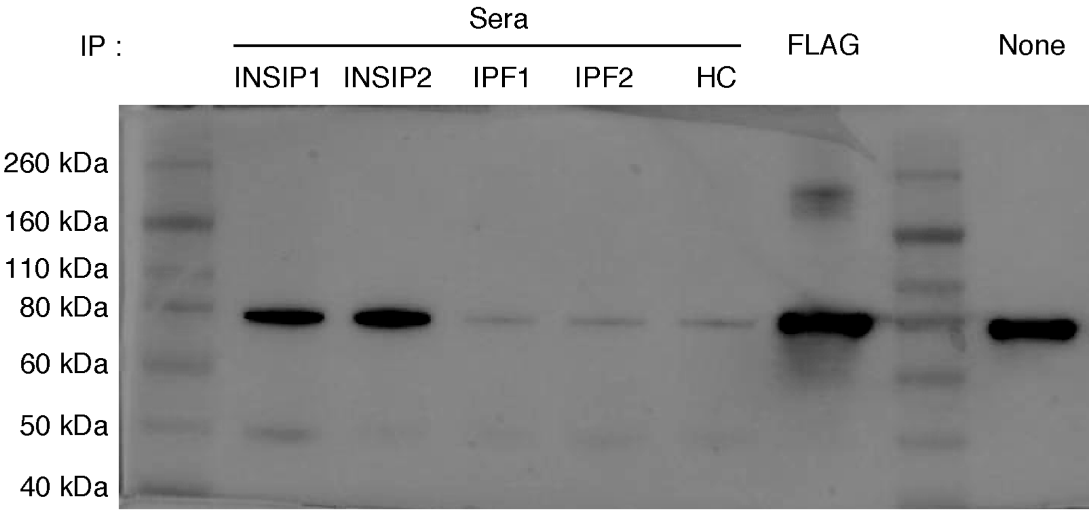

Supplement: Supplementary Information [file srep43201-s1.pdf]
